# Insights into the Transcriptome of Human Cytomegalovirus: A Comprehensive Review

**DOI:** 10.3390/v15081703

**Published:** 2023-08-08

**Authors:** Janine Zeng, Di Cao, Shaomin Yang, Dabbu Kumar Jaijyan, Xiaolian Liu, Songbin Wu, Ruth Cruz-Cosme, Qiyi Tang, Hua Zhu

**Affiliations:** 1 Department of Microbiology and Molecular Genetics, New Jersey Medical School, Rutgers University, 225 Warren Street, Newark, NJ 070101, USA; 2 Department of Pain Medicine, Huazhong University of Science and Technology Union Shenzhen Hospital, Shenzhen 518052, China; 3 Institute of Pathogenic Organisms, Shenzhen Center for Disease Control and Prevention, Shenzhen 518055, China; 4 Department of Microbiology, Howard University College of Medicine, 520 W Street NW, Washington, DC 20059, USA

**Keywords:** HCMV, transcriptome, mRNA, lncRNA, circRNA, miRNA

## Abstract

Human cytomegalovirus (HCMV) is a widespread pathogen that poses significant risks to immunocompromised individuals. Its genome spans over 230 kbp and potentially encodes over 200 open-reading frames. The HCMV transcriptome consists of various types of RNAs, including messenger RNAs (mRNAs), long non-coding RNAs (lncRNAs), circular RNAs (circRNAs), and microRNAs (miRNAs), with emerging insights into their biological functions. HCMV mRNAs are involved in crucial viral processes, such as viral replication, transcription, and translation regulation, as well as immune modulation and other effects on host cells. Additionally, four lncRNAs (RNA1.2, RNA2.7, RNA4.9, and RNA5.0) have been identified in HCMV, which play important roles in lytic replication like bypassing acute antiviral responses, promoting cell movement and viral spread, and maintaining HCMV latency. CircRNAs have gained attention for their important and diverse biological functions, including association with different diseases, acting as microRNA sponges, regulating parental gene expression, and serving as translation templates. Remarkably, HCMV encodes miRNAs which play critical roles in silencing human genes and other functions. This review gives an overview of human cytomegalovirus and current research on the HCMV transcriptome during lytic and latent infection.

## 1. Introduction

The human cytomegalovirus (HCMV) belongs to the beta-herpesvirus subfamily and is a double-stranded DNA virus, infecting 40% to 60% of individuals in industrialized countries and up to 100% in developing countries. It is transmitted through body fluids, blood transfusion, and organ transplantation. While mostly asymptomatic in immunocompetent individuals, HCMV can remain latent after primary infection and reactivate during pregnancy or in individuals with cancer, transplanted organs, AIDS, or other immune deficiencies [1], leading to severe diseases in the lung, liver, colon, eye, or brain such as hepatitis, pneumonitis, colitis, and CMV retinitis [1]. Additionally, congenital CMV (cCMV) infection is a leading cause of birth defects, with approximately 10% of infants with cCMV displaying CNS impairments [2].

The mature HCMV virion contains a large linear double-stranded genomic DNA tightly intertwined and wrapped within a capsid, which is surrounded by a tegument layer and an envelope [3]. The HCMV genome is approximately 230 to 240 kbp in length, comprising over 200 open-reading frames (ORFs) [4] that serve different functions such as for HCMV survival, replication, and infection.

**Figure 1:**
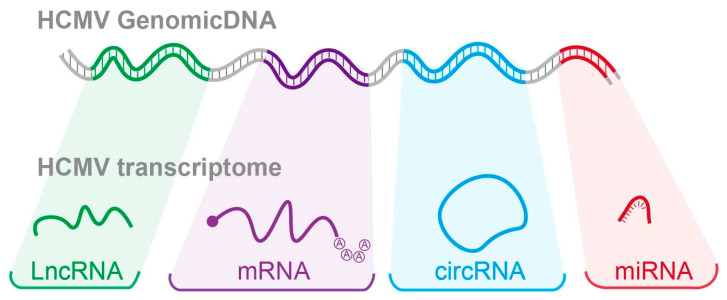
An overview of HCMV transcriptome.

The HCMV transcriptome includes polyadenylated (polyA) protein-coding and polyA non-protein coding RNAs. Notably, 65.1% of poly A viral RNA transcription produces four long non-coding RNAs (lncRNAs). Furthermore, the HCMV transcriptome contains other types of non-coding RNAs, such as microRNAs, lncRNAs [5], and the recently discovered circular RNAs (circRNAs) [6] and microRNAs (miRNAs) [7] (Figure 1).

While the functions of some HCMV genes remain unknown, significant progress has been made in identifying the functions of genes related to the infective stages [8]. In this review, we provide an overview of current research on the four different classes of HCMV transcripts and delve into their respective roles and functions.

## 2. HCMV Messenger RNA (mRNA)

**Figure 2:**
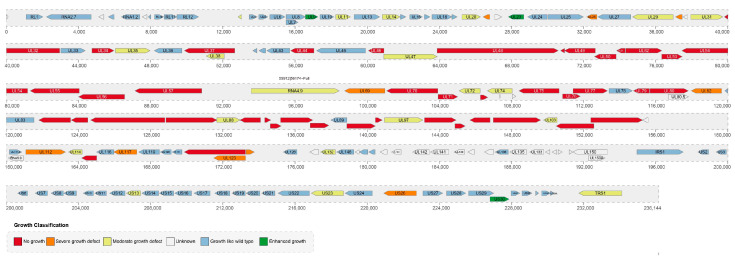
Genome organization of HCMV.

The human cytomegalovirus (HCMV) was first discovered in 1881 [9]. Early sequencing and annotation of the laboratory strain of HCMV AD169 sequenced around 208 ORFs [10] (Figure 2), but subsequent re-evaluation estimated the number of protein-coding sequences to range from 164 to 220 [11,12,13,14,15,16]. Among these, 45 ORFs were found essential for viral replication in fibroblasts, while 107 were deemed nonessential [16]. However, recent studies have identified over 400 newly translated ORFs by ribosome profiling, bringing the total number to over 750 with many transcripts containing multiple translationally active ORFs [17]. Subsequently, a comprehensive analysis reported 248 transcription start sites, 116 transcription termination sites, and 80 splicing events within the HCMV genome. Furthermore, 291 previously undescribed or only partially annotated transcription isoforms were identified and annotated. Most of these transcripts were found to contain multiple translationally active ORFs [15], adding to the complexity of HCMV gene expression and regulation.

### The ORFs Are Color-Coded According to the Growth Properties of Their Corresponding Virus Gene Deletion Mutants

**Figure 3:**
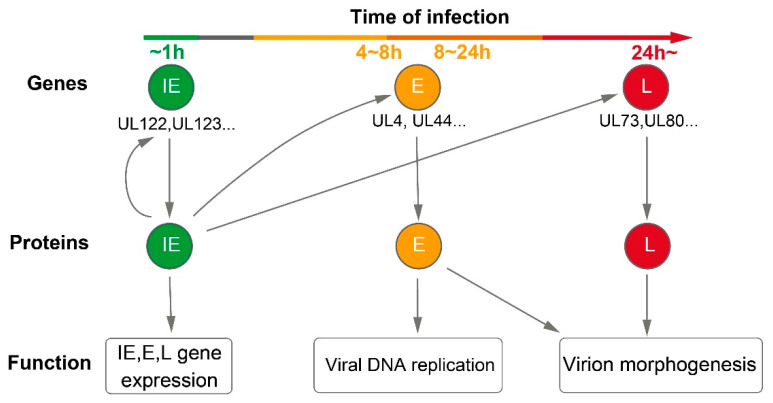
HCMV gene expression during productive infection.

The expression of HCMV genes is temporally regulated, and it is divided into immediate early (IE), early (E), and late (L) gene expression [18] (Figure 3). IE genes encode regulatory trans-acting factors, while the E genes’ expression requires the de novo expression of IE genes. Late gene expression occurs after the onset of viral DNA replication [19,20]. Due to the complexity of the HCMV genome, the roles and protein-coding potentials of many ORFs remain largely unknown, warranting further investigation. Some major ORFs functions identified include protein coding, viral replication, and translation regulation.

Research has identified over 30 ORFs that are vital for viral replication. For example, the de novo synthesis of pUL21A promotes the synthesis of viral DNA, which is required for the late accumulation of IE transcripts and establishment of productive infection [21,22] (Figure 2). UL123-coded 72-kDa IE1 also promotes viral replication and transcription by antagonizing histone deacetylation, whereas pUL76 has a dominant-negative effect on replication [23]. Furthermore, some HCMV genes are involved in the viral translation process. The HCMV gpUL4 mRNA contains a 22-codon upstream open-reading frame (uORF2) whose product represses downstream translation by blocking translation termination and causing ribosomes to stall on the mRNA [24,25]. HCMV pUL38 preserves mTORC1 kinase activity that promotes translation initiation [26]. Moreover, pUL38 and pUL69 support translation by antagonizing the mTOR target 4EBP1 [27]. PTRS1 enhances translation through both PKR-dependent and PKR-independent mechanisms, limiting the host’s antiviral response [28].

In addition to their role in targeting the virus itself, HCMV genes exert various effects on host cells, including immune regulation, cell apoptosis, and proliferation (Table 1). Notably, approximately 58 genes have been identified to be involved in immune regulation, enabling the virus to evade host antiviral responses. For instance, UL18 inhibits the cytolytic activity of NK cells and the production of inflammatory cytokine IFN-γ through the ILT-2 receptor, thereby evading NK cell-mediated cytotoxicity [29]. Another set of genes, including pUL7 and the miRNAs miR-US5-1 and miR-UL112-3p, are implicated in restricting the expression and activation of the transcription factor FOXO3a, leading to the prevention of virus-induced apoptosis in CD34+ hematopoietic progenitor cells [30]. Additionally, the gene pUL36 interferes with the death ligand-mediated apoptotic pathway at an upstream step of caspase 8 activation [31]. Certain genes, such as UL82, UL128 [32], US27, IE1 [33,34], and IE2 [34] could induce cell proliferation, while others like UL10 [35], UL11 [36], and UL144 [37] inhibit cell proliferation. A comprehensive summary of the functions of each gene involved in the regulation of host cells is shown in Table 1, which has been compiled and updated based on the review by Mocarski (2007) and Damme (2014) along with subsequent research exploring the functions of HCMV genes [38,39]. These findings emphasize the complex interplay between HCMV and the host immune system and provide valuable insights into the mechanisms of viral evasion and their impact on host cellular processes.

## 3. HCMV Long Non-Coding RNAs (lncRNAs)

Long non-coding RNAs (lncRNAs) are a class of transcripts that consist of over 200 nucleotides but do not encode proteins. Within the context of HCMV, four main lncRNAs, namely RNA1.2, RNA2.7, RNA4.9, and RNA5.0, account for over 50% of the poly (A)+ viral transcriptome in all infection states (Table 2). Among these lncRNAs, RNA1.2, RNA2.7, and RNA4.9 have been found to play important roles in the overall HCMV viral life cycle, particularly during lytic replication [5]. However, further investigation is still required to fully understand the detailed functions of lncRNAs in HCMV. Here, we aim to provide a comprehensive summary of the existing research on the four HCMV long non-coding RNAs and their potential contributions to the virus’s replication.

### 3.1. RNA1.2

RNA1.2 is among the earliest HCMV transcripts to be discovered and accounts for approximately 7.9% of viral polyA RNA transcription [12]. Although many of its functions are still unknown, it is likely that lncRNA1.2 does not play a large role in the main processes of viral production, such as entry, genome replication, virion assembly, and egress. However, research has indicated that RNA1.2 does have important functions in modulating the expression of multiple cellular genes and facilitating the evasion of acute antiviral responses. One of RNA1.2’s roles involves downregulating TPRG1L, which in turn blocks NF-κB and suppresses both the expression and secretion of proinflammatory mediators like IL-6 [43]. As a result, further investigation into RNA1.2 could potentially contribute to the development of treatments for IL-6-associated illnesses. Additionally, lncRNA1.2 generates multiple natural antisense transcripts (NATs) during the late infection stage of HCMV. While it has been found that RNA1.2 ASTs (antisense transcripts) play a role in regulating sense strand expression, further research is necessary to determine the importance of RNA1.2 ASTs in the regulation of the expression of the RNA1.2 gene [44].

### 3.2. RNA2.7

The HCMV lncRNA2.7 is the most abundant lncRNA, occupying approximately 29% of the total poly (A) viral transcriptome [5]. Extensive research indicates that this lncRNA has an important role during infection, particularly in promoting the movement and detachment of infected cells during late infection [517]. Specifically, RNA2.7 facilitates cell movement and viral spread during late infection by stabilizing mRNAs that are rich in A and U nucleotides. It also regulates a large number of cellular genes late in the lytic infection, many of which are associated with encouraging cell movement [517]. Additionally. RNA2.7 has been shown to increase cell-to-cell viral transmission, which is likely because of its role in facilitating cell movement [517]. Moreover, research suggests that RNA2.7 may also be involved in the processes related to latency or reactivation, such as cellular transcription and cell cycle progression. Additionally, it contributes to boosting viral replication by reducing the host’s response to infection through repressing Pol II S2 phosphorylation [42]. While some functions and roles of lncRNA2.7 have been revealed, like other HCMV lncRNAs, further research is still required to fully understand its importance and the intricate mechanisms by which it influences various aspects of the viral life cycle.

### 3.3. RNA4.9

Unlike the other three HCMV lncRNAs, which are predominantly localized in the cytoplasm, RNA4.9 is uniquely distributed in the viral replication compartment. RNA4.9 is transcribed in this compartment during early infection, and its levels increase as infection progresses [192]. One notable feature of RNA4.9 is its ability to form RNA–DNA hybrids (R-loops) through its G+C rich region. This interaction may be involved with the initiation of DNA replication [518], as a reduction in RNA4.9 expression correlates with decreased viral DNA replication. This finding strongly suggests that RNA4.9 plays a direct role in viral DNA replication and growth [192]. In addition to its role in viral DNA replication, RNA4.9 may be involved in HCMV latency. The lnc4.9 RNA has been found to associate with the polycomb repressor complex 2 (PRC2) [519]. In herpes simplex virus (HSV), PRC2 plays a role in regulating viral latency [191]. This association raises the possibility that RNA4.9 might play a role in HCMV latency as well. Further research on whether virus mutants that do not express RNA4.9 fail to establish and maintain latency could provide more insight into the role of RNA4.9 in HCMV latency [520].

### 3.4. RNA5.0

The lncRNA5.0 is a stable intron expressed during HCMV infection that is transcribed by RNA polymerase II and characterized by a high adenine and thymine nucleotide content [5,521]. However, compared to other HCMV lncRNAs, the expression of RNA5.0 is much lower than other HCMV lncRNAs, accounting for approximately 0.1% of the total viral transcriptome, and it is not present in the poly (A)+ viral transcriptome since it does not contain a poly (A) tail [5]. RNA5.0 is primarily localized in the nucleus during viral infection and lacks potential protein-coding ORFs [521]. While the exact functions of RNA5.0 remain largely unknown, research suggests that RNA5.0 may not be necessary for lytic replication and the maintenance of latent reservoirs, unlike the HCMV lncRNAs RNA1.2, RNA2.7, and RNA4.9 [5]. However, despite its relatively low expression and lack of its exact functions, lnc5.0 RNA may play a role in activating transcription, regulating gene silencing, or impacting HCMV latency. It could also function in an important role like immune evasion that is important for infection of host organisms but not in cultured cells [521]. Given the limited knowledge about lnc5.0 RNA’s precise functions, further investigation is necessary to elucidate its role in HCMV infection fully.

## 4. HCMV Circular RNAs (circRNAs)

Circular RNAs (circRNAs) are a unique class of RNA molecules formed through back splicing, resulting in covalently closed loops that lack a 5′ cap and a 3′ poly (A) tail [522]. Due to their circular structure, circRNAs are more resistant to exoribonuclease (such as RNase R) than linear RNAs [522]. CircRNAs have been identified in all kinds of cells and demonstrated to be associated with different diseases, indicating that they possess important biological functions. CircRNAs can function as microRNA (miRNA) sponges, regulate of parental gene expression, and even serve as translation templates [522,523]. They have also been identified from DNA virus-infected cells, such as Epstein–Barr virus (EBV) [524,525,526,527], Kaposi Sarcoma herpesvirus (KSHV) [525,528,529,530], human papillomaviruses (HPVs) [531] and RNA viruses, severe acute respiratory disease coronavirus 2 (SARS-CoV-2) [532] and murine hepatitis virus (MHV) [533]. This suggests that circRNAs may play important roles in the viral life cycle and infection processes.

**Figure 4:**
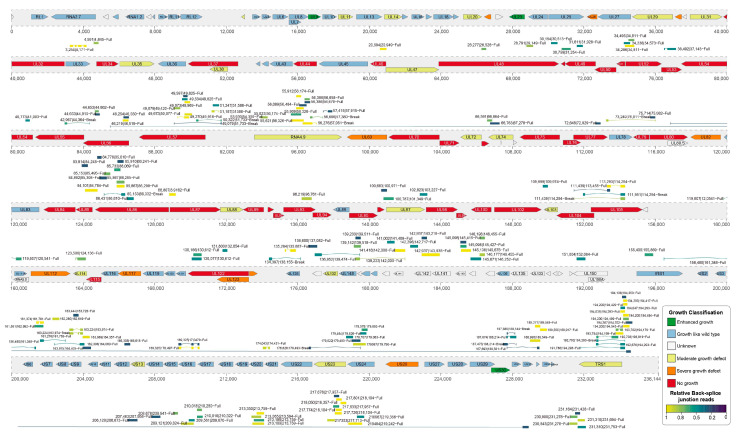
Genome organization and full-length circRNAs of HCMV. The ORFs are color-coded according to the growth properties of their corresponding virus gene-deletion mutants. Variation multigene segment of the HAN strain: ~15 kb (176,729 to 191,263 bp). The relative expression levels of circRNAs are indicated by the colors at the bottom. Reconstruction of full-length circRNAs and partially assembled circRNAs are indicated by “Full” and “Break”, respectively.

In our previous study, we bioinformatically predicted 704 candidate circRNAs encoded by the HCMV TB40/E strain and 230 encoded by the HCMV HAN strain (Figure 4) [6]. Furthermore, we experimentally confirmed 324 back-splice junctions (BSJs) from three HCMV strains, Towne, TB40/E, and Toledo. A newly published work by Deng et al. also experimentally confirmed 629 HCMV circRNAs from the HAN strain [534]. More importantly, we found 12 circRNAs with over-alignment lengths of 40 bp from 60 bp BSJ sequences that were conserved in both the HAN and TB40/E strains and also expressed in several cell types, suggesting these circRNAs are selected and play important roles (Table 3). Functional analysis of HCMV circRNAs in a competitive endogenous RNA co-regulatory network shows that HCMV circRNAs are involved in a complex and multifaceted interaction network. CircRNAs are an important component of the HCMV transcriptome, and further mutagenesis studies on HCMV circRNA biogenesis may reveal the role played by HCMV circRNAs in terms of viral replication, latency, reactivation and host cells.

## 5. HCMV microRNA (miRNA)

HCMV microRNAs (miRNAs) are small non-coding RNA molecules that consist of approximately 22 nucleotides and distributed throughout the HCMV genome. They account for around 80% of total HCMV reads obtained from deep sequencing [7,535]. HCMV encodes 17 known mature miRNAs from 11 precursors (Table 4). In addition, recent research has identified 10 new HCMV miRNAs, 4 from known precursors and 6 from new precursors, bringing the total number of mature miRNAs to 22 from 13 different precursors [20,535,536]. The high expression of miRNAs in the HCMV genome also suggests that they play an important biological role during infection [535].

Viral miRNAs from other herpesviruses like EBV and HSV have promoted the establishment and maintenance of latency. HCMV miRNA may have similar functions [537]. Additionally, miRNAs are non-immunogenic and capable of targeting multiple cellular and viral transcripts, providing an effective means for HCMV to manipulate viral gene expression and cellular signaling pathways during both lytic and latent infection [538]. By targeting numerous cellular genes and modulating the host’s signaling pathways, HCMV miRNAs contribute to viral survival and replication [7]. HCMV miRNAs can also silence human genes involved in various physiological processes and attenuate the expression of immediate early (IE) proteins, which are vital for lytic replication. Overall, miRNAs are an important component of the HCMV genome.

Furthermore, research suggests that HCMV miRNAs have the potential to be involved in the development and progression of human diseases [7]. For instance, HCMV miR-US33-5p was found to influence the apoptosis of human aortic vascular smooth muscle cells (HA-VSMC) and was more abundant in the plasma of patients with acute aortic dissection (AAD) [539]. This indicates that HCMV miRNAs might have implications in the pathogenesis of certain human diseases, offering new possibilities for potential treatment alternatives. Understanding the functions and roles of HCMV miRNAs not only provides valuable insights into how the virus operates but also opens up new avenues for exploring therapeutic strategies for HCMV-associated diseases. Further research in this area may reveal novel targets for intervention and management of HCMV infections and related health conditions.

**Table 4:** The information of conserved HCMV miRNAs during lytic infection.

miRNA	Sequence	Start	End	Length
hcmv-miR- UL22A-5p	TAACTAGCCTTCCCGTGAGA	27,992	28,011	19
hcmv-miR- UL22A-3p	TCACCAGAATGCTAGTTTGTAG	28,029	28,050	21
hcmv-miR- UL36-5p	TCGTTGAAGACACCTGGAAAGA	49,914	49,893	21
hcmv-miR- UL36-3p	TTTCCAGGTGTTTTCAACGTG	49,870	49,851	19
hcmv-miR- UL70-5p	TGCGTCTCGGCCTCGTCCAGA	104,404	104,424	20
hcmv-miR- UL70-3p	GGGGATGGGCTGGCGCGCGG	104,445	104,464	19
hcmv-miR- UL112-3p	AAGTGACGGTGAGATCCAGGC	164,557	164,578	21
hcmv-miR- UL148D	TCGTCCTCCCCTTCTTCACCG	193,587	193,607	20
hcmv-miR- US4-5p	TGGACGTGCAGGGGGATGTC	201,376	201,395	19
hcmv-miR- US5-1-3p	TGACAAGCCTGACGAGAGCGT	202,317	202,337	20
hcmv-miR- US5-2-3p	TTATGATAGGTGTGACGATGTC	202,444	202,465	21
hcmv-miR- US25-1-5p	AACCGCTCAGTGGCTCGGACC	221,539	221,519	20
hcmv-miR- US25-1-3p	TCCGAACGCTAGGTCGGTTCT	221,496	221,476	20
hcmv-miR- US25-2-5p	AGCGGTCTGTTCAGGTGGATGA	221,760	221,739	21
hcmv-miR- US25-2-3p	ATCCACTTGGAGAGCTCCCGCGG T	221,702	221,680	22
hcmv-miR- US33-5p	GATTGTGCCCGGACCGTGGGCG	226,768	226,750	18
hcmv-miR- US33-3p	TCACGGTCCGAGCACATCCA	226,731	226,712	19

MiRNAs were downloaded from miRbase database, which is a searchable database of published miRNA sequences and annotation [540].

## 6. HCMV Gene Expression during a State of Latency

HCMV establishes latency primarily in early myeloid lineage cells [541,542], such as CD14+ monocytes and CD34+ hematopoietic progenitor cells [543]. The transcriptome of latent HCMV is very challenging to define, in part because of the scarcity of latently infected cells and the lack of a suitable model. The fate of the virus is determined by the type of infected cells, where the infection of fibroblast cells leads to the production of infectious progeny virus, while the infection of myeloid progenitor cells leads to virus latency, which is acharacterized by the maintenance of the viral genome in the absence of active virus infection or replication. The molecular mechanisms governing viral latency are poorly understood. It has been reported that the HCMV transcriptome during latency is qualitatively different from the lytic cycle transcription profile [544]. Studies using a virus gene-specific microarray have identified latency-associated genes in HCMV-infected myeloid progenitor cells [545,546]. Additionally, using nested PCR, researchers have identified several viral genes with distinct transcriptional profiles during virus latency [544,547]. The transcriptomic profiling of HCMV-infected CD34+ cells and CD14+ monocytes led to the identification of around 20 genes that were associated with latent viral infection [191]. Moreover, the single-cells transcriptomic profiling of latently infected monocytes found a cellular heterogeneity in response to latent virus infection [548,549].

A number of genes including UL138 and LUNA are present during latent virus infection [191,550]. Other genes such as UL144, the IE1 region, UL111A, US28, and non-coding RNAs 4.9 and 2.7 were also expressed during the lytic as well as latent virus phases [322,546,551,552]. It has been hypothesized that lytic genes are expressed during an early phase of viral latency and then repressed over time [553]. Furthermore, research suggests that the heterochromatinization of viral DNA takes place to repress gene transcription during latency. Some studies have shown that signaling pathways mediated through platelet-derived growth factors (PDGFR), epidermal growth factor (EGFR), and PI3K along with the downregulation of IE1/2 expression, UL138 upregulation, and perturbation of cytokine expression leads to viral latency [554,555,556]. In addition to viral genes, HCMV-encoded miRNAs have been shown to have important roles in the establishment of latency. They include miR-UL148D and miR-UL112-1. Another miRNA, has-miR-s200, was also found to play an important role in HCMV latency [557].

It has been reported that MIEP is the master regulator of latency in infected cells. In latently infected cells, MIEP is heterochromatinized, suggesting a latency-specific function. Several transcription factors including Elk-1, NF-κB, SRF, AP-1, CREB, and Sp1 have binding sites in MIEP and thus play roles in virus latency either directly or indirectly [18,558,559]. The accumulating evidence suggests that the transcriptomic profiling of latent HCMV has heterogeneity and is poorly defined. Moreover, the exact cause of transcriptional repression of virus gene transcription during latency is unclear, and the involvement of other viral and cellular factors in the establishment of virus latency needs to be identified to better understand this complex process.

## 7. Conclusions

Indeed, human cytomegalovirus (HCMV) infection can vary greatly depending on the individual’s immune status. While it remains latent and asymptomatic in many healthy individuals, HCMV poses a significant health risk for those who are immunocompromised, such as transplant recipients, HIV patients, and infants with congenital infections. Research on the HCMV transcriptome, including mRNAs, lncRNAs, circRNAs, and miRNAs, has provided valuable insights into the complex interactions between the virus and its host. These different types of RNAs play diverse and overlapping functions in HCMV infection, contributing to various aspects of the virus life cycle, including replication, latency, reactivation, immune regulation, protein coding, and cell movement. Despite significant progress in understanding the HCMV transcriptome, there are still areas of the HCMV transcriptome that are not fully investigated. Further research on HCMV pathogenesis and its transcriptome may lead to a better understanding of human cytomegalovirus as well as insights into effective treatments for HCMV diseases. This knowledge can potentially lead to the development of more effective treatments for HCMV-related diseases, especially for immunocompromised patients and infants at risk of congenital infections.

## Figures and Tables

**Table 1:** The potential function of HMCV ORFs.

ORFs	Detail Functions	Functions to Host Cells
RL1	Degrades SLFN11 [40]	Immune evasion [40]
RNA2.7 (IRL4)	Lytic replication and maintenance of latent [5]; Inhibits apoptosis, maintains ATP production [41]; Inhibits Pol II phosphorylation [42]	Anti-apoptosis [41] Cell transcription [42]
RL5A	Unknown	Unknown
RL6	Unknown	Unknown
RNA1.2	Lytic replication and maintenance of latent [5]; Suppresses upregulation of IL-6 by blocking NF-κB activation [43]; Transcripts a new NAT [44]	Immune evasion [43]
RL8A	Unknown	Unknown
RL9A	Unknown	Unknown
RL10	Unknown	Unknown
RL11	IgG Fc-binding protein, blocks IgG-mediated activation of FcγRs [45,46]; Induces early ADCC [47]	Immune evasion [46]
RL12	IgG Fc-binding protein capacity [48]	Unknown
RL13	IgG Fc-binding protein [48]; Inhibitor of replication [49]; Interacts with NUDT14 [50]	Immune evasion [48]
UL1	Cell type-specific tropism factor [51]	Cell tropism [51]
UL2	Unknown	Unknown
UL4	Represses translation, ribosome stalling [24,25,52]	Unknown
UL5	Induces early ADCC [47]; Interacts with IQGAP1 [53]	Immune evasion [47]
UL6	Unknown	Unknown
UL7	Reduces FOXO3a activity, prevents apoptosis in CD341HPCs [30]; Interferes with proinflammatory responses [54]; CEACAM1-like protein, promotes angiogenesis [55]; Binds to Flt3R triggers HPC and monocyte differentiation [56]	Anti-apoptosis [30] Anti-inflammatory [54] Promotes angiogenesis [55]
UL8	Inhibits the production of proinflammatory cytokine [57]	Anti-inflammatory [57]
UL9	Temperance for fibroblasts [38]	Unknown
UL10	Interference of T cells activation, proliferation, and cytokine production [35]; Temperance for RPE [38]	Anti-cell proliferation, immune evasion [35]
UL11	Inhibits T cell signaling and proliferation via CD45 [36]; Induces T cell IL-10 secretion via CD45 [58]	Anti-inflammatory, Anti-cell proliferation [36,58]
UL12	Unknown	Unknown
UL13	Unknown	Unknown
UL14	Unknown	Unknown
UL15A	Unknown	Unknown
UL15	Unknown	Unknown
UL16	Induces early ADCC [47]; Temperance in fibroblasts [38]; Modulation NK cell signaling/function [39,59,60,61,62]	Immune evasion [39,47,59,60,61,62]
UL17	Unknown	Unknown
UL18	MHC-I homologue, LIR-1 ligand, modulation NK cell signaling [29,39,63,64,65,66,67]	Immune evasion [29,39,63,64,65,66,67]
UL19	Unknown	Unknown
UL20	Targets lysosomal degradation [68]	Unknown
UL21	Unknown	Unknown
UL21A (UL21.5)	Facilitates virus replication and late gene expression [21,22]; APC regulator [69,70]; Degradation of cyclin A, proteasomal destruction [71,72]	Cell cycle arrest [71,72]
UL22A (UL22.5)	Secretory CCL5 receptor [73]; Selective RANTES binding [74]	Immune evasion [74]
UL22	Unknown	Unknown
UL23	Temperance for fibroblasts [38]; Inhibits IFN-γ/IFN-I response [75,76,77]	Immune evasion [75,76,77]
UL24	Downregulates the expression of miR-UL59 [78]; Participates in SAMHD1 subcellular localization [79]	Immune evasion [78]
UL25	Presumably binds to the SH3 domain of NCK-1 [80]	Unknown
UL26	Activator of MIEP [81]; Regulates the phosphorylation of pp28 [82]; Viral tegument assembly [83]; Increases stability of virion proteins [84,85]; Antagonize innate immunity (via NF-κB signaling) [86,87]	Immune evasion [86,87]
UL27	Maribavir resistance [88,89,90,91]	Unknown
UL28	Activator of MIEP (via NuRD complex) [92,93]; Regulates p53 transcriptional activity [94]	Unknown
UL29	Activator of MIEP (via NuRD complex) [92,93]; Regulates p53 transcriptional activity [94]	Unknown
UL30	Unknown	Unknown
UL31	Regulate pre-rRNA levels and nucleolar organization [95]; Inhibitor of cGAS [96]	Immune evasion [96]
UL32 (PP150)	Virion maturation, nucleocapsid stabilization [97,98,99]; Cyclin A2–CDK-dependent sensor [100]; Viral DUB main target [101]	Cell cycle arrest [100]
UL33	Orchestrates signaling networks [102]; Modulates chemokine receptors [103,104]; Oncomodulatory properties [105]; Facilitates cell-associated and cell-free spread [106]; Activates CREB, viral reactivation, trophoblast migration [107,108]	Chemokine receptor [103] Cell migration [108]
UL34	Represses US3 expression [109,110]; Transcriptional repression and oriLyt-dependent DNA replication [111,112,113]; Capsid formation and maturation [114]	Unknown
UL35	IE gene expression and virus assembly [115,116]; Promotes viral replication by manipulating host responses [117]; Antagonizes type I IFN response [118]	Immune evasion [118]
UL36	Regulators gene expression [119]; Inhibits caspase-8 activation and apoptosis [31,120,121]; Initiation of replication [122]	Anti-apoptosis [31,120,121]
UL37.1	Regulators gene expression [119]; Initiation of replication [122]; US3 regulator (repressor) [123]; Viral mitochondrion-localized inhibitor of apoptosis (vMIA) [124,125,126,127]; Important for remodeling of host lipid metabolism [128]	Immune evasion, anti-apoptosis [124,125]
UL37.3	Initiation of replication [122]; MHC-like protein [129]; Prerequisites for gpUL37 internal cleavage [130]	Immune evasion [129]
UL38	US3 regulator (repressor) [123]; Anti-apoptotic, facilitates virus replication [26,119,131,132,133]; Regulates p53 transcriptional activity [94]; Induces a pro-viral metabolic environment (via inhibition of TSC2) [134]	Anti-apoptosis [26,119,131,132,133]
UL39	Unknown	Unknown
UL40	Regulator of NK cell signaling [135,136,137,138]	Immune evasion [135,136,137,138]
UL41A (UL41.5)	Unknown	Unknown
UL42	Negative regulator of cGAS/MITA [139]; Inhibition of E3 ligase prevents gB degradation [140,141]; Activates c-Jun [142]	Immune evasion [139]
UL43	Downregulates the expression of miR-UL59 [78]	Immune evasion [78]
UL44	DNA-binding nuclear protein, complexed to nucleolin and dsDNA [143]; Natural substrate of UL97 [144]; DNA polymerase accessory protein, increases DNA Pol processivity [145,146,147]; Inhibits p53 transcriptional activity [148]; Inhibits the binding of IRF3 and NF-κB to promoters of antiviral genes [149]; Sumoylation of UL44 attenuates viral replication [150,151]	Immune evasion [149]
UL45	Late virion-associated protein [152]; Homologue of RNR R1, inhibits NF-κB signaling [153,154,155]	Immune evasion [153]
UL46	Capsid constituent [156]	Unknown
UL47	Modulates tegumentation and capsid accumulation [157]; Releases viral DNA from capsid [84,158]	Unknown
UL48	Homologue of RNR R1, inhibits NF-κB signaling [153]; Deubiquitin protease [159]; Regulates the localization of pUL47 in vAC and promotes capsid maturation [84,157]; Releases viral DNA from capsid [158]; Contributes to vAC biogenesis [160]; Contributes to viral growth, virion stability and virus entry [161]	Immune evasion [153]
UL48A (UL48.5)	Smallest capsid protein [162]; Locates on tips of hexons in capsids [163]	Unknown
UL49	DNA replication [164,165,166]; Regulates late-stage gene transcription, vPIC member [167]	Unknown
UL50	Recruits UL97, nuclear egress, disruption of nuclear lamina [168]; Negative regulator of protein ISGylation [169]; Recruits UL53 to the nuclear membrane [170]; Induces the loss of VCP/p97 [171]	Unknown
UL51	DNA cleavage packaging [172]; Viral terminase, promotes nuclear localization [173,174]	Unknown
UL52	DNA cleavage packaging [175]	Unknown
UL53	Recruits UL97, nuclear egress, disruption of nuclear lamina [168,176]; Functional virion production [177]; Associates with capsids and myosin Va [178]	Unknown
UL54	DNA polymerase catalytic subunit (POL) [38]; Drug resistance [179]; Increases DNA processivity [145,146,147]	Unknown
UL55	Virion glycoprotein B (gB), virion penetration, cell fusion and spread [180,181,182,183]; Formation of replication compartment [184]; Heparan binding [38,185]	Cell spread and fusion [180,181,182,183]
UL56	Formation of replication compartment [184]; Binds DNA packaging motif, DNA cleavage, nuclease activity [38,186]; Terminase subunits [172,173,187]; ATPase activity [188]	Unknown
UL57	Formation of replication compartment [184]; ssDNA-binding protein (SSB) [189]	Unknown
RNA4.9	Viral latency, binds to PRC2 [190]; Enrichment of the repressive H3K27me3 mark [191]; Regulates viral DNA replication [192]	Cell proliferation [190]
UL69	Pleiotropic transactivator [193]; Facilitates translation [27]; Promotes nuclear export of unspliced RNA [194]; Induces cell cycle block [195,196]; Nucleocytoplasmic shuttling activity [197]; mRNA export and viral replication [198,199]	Cell cycle arrest [195,196]
UL70	Primase [189,200]; Viral DNA synthesis, progeny production [201,202]	Unknown
UL71	Virus spread and release, affects multivesicular bodies [203]; Secondary envelopment [204,205,206]	Unknown
UL72	Transcription-replication machinery [4]; Deoxyuridine triphosphatase homologue (not active) [207]	Unknown
UL73	Envelope component [208]; Virion glycoprotein N (gN), component of gC-II, intracellular transport [209,210]; Virus attachment and cell spread [211]	Cell spread [211]
UL74	Virion glycoprotein (gO), gCIII envelope complex components [212]; Cell fusion [213,214,215]; Promotes secondary envelopment and virus release [216]; Inhibits gH and gB antibodies, promotes focal spread [217]; Promotes gH/gL into the virion [218]; Polymorphisms, antibody neutralization on gH epitopes [219]	Cell fusion [213,214,215]; Immune evasion [217]
UL74A	Unknown	Unknown
UL75	Virion glycoprotein (gH), cell fusion [214]; Viral entry, activates gene expression (Sp1 and NF-kappaB) [180,220]	Cell fusion [214]
UL76	Modulates gene expression, inhibits DNA replication [23]; Induces DNA damage and chromosome aberrations [221,222]; Regulates UL77 expression [223]; Aggresome formation [224]	Unknown
UL77	Capsid maturation, DNA packaging [225]; DNA binding capacity [226]	Unknown
UL78	GPCR homologues, chemokine receptor-like protein [103]; Regulates chemokine receptor [104]; Enters cells and delivers virion components [227]; Negatively regulates NF-κB pathway [228]	Immune evasion [228]
UL79	The accumulation of late viral transcripts [229]; Late viral gene expression, infectious virus production [230]; Nuclear-accumulating protein, involved in nuclear import pathway [231]	Unknown
UL80	Serine protease and its substrate [232]; Capsid assembly proteins, involved in nuclear localization signals, virus production [233]	Unknown
UL80.5	Assembly protein precursor (pAP), interacts with MCP via CCD [234,235]	Unknown
UL80A	Maturational protease precursor (pPR) [233]; Early capsid formation, interacts with itself through ACD [236]	Unknown
UL82 (PP71)	Activator of MIEP [237,238]; Stimulates G1 cell cycle progression, induces cell DNA synthesis [239,240]; Binds and degrades Rb family members [240,241,242]; Regulates IE gene expression and viral replication [243,244]; Interferes with trafficking and cell surface expression of MHC class I [245]; Induces Daxx SUMOylation [246,247]; Inhibits STING-mediated signaling, evades antiviral immunity [248]	Cell proliferation [190] Cell cycle stimulation [239,240] Immune evasion [244,247,248]
UL83 (PP65)	Modulates immune response [249]; Prevents IRF3 activation, attenuates the interferon response [250]; Inhibits NK cell activity [251,252]; Degrades HLA-DR α-chain [253,254]; Modulates viral gene expression and IFI16 stability [255,256,257]; Deregulates the activation of AIM2 inflammasome [258]; Inhibits IFN-β production via cGAS [259]	Immune evasion [249,250,254,256,257,258,259]
UL84	Suppresses the transcriptional activation of IE2, UTPase activity [200]; OriLyt-dependent DNA replication, activates oriLyt promoter, initiates lytic replication [260,261,262,263,264]; Component of viral replication compartments [265]; Nucleocytoplasmic essential for viral growth [266,267]; Enhances p53 binding [268]	Unknown
UL85	Minor capsid protein [38,156]	Unknown
UL86	Major capsid protein [38,235,269]	Unknown
UL87	Late gene expression, inhibits MIE genes expression and virus DNA replication [191,230]	Unknown
UL88	Maintains the virion proper tegument composition [270]	Unknown
UL89	Terminase ATPase subunit, promotes terminase complex formation [172,173]; Effects DNA cleavage, produces energy for genome transportation [271,272]; Viral maturation [273,274,275]	Unknown
UL90	Unknown	Unknown
UL91	Regulates late viral gene expression [276]	Unknown
UL92	Regulator of late viral gene expression [277]	Unknown
UL93	Interacts with pUL77, necessary for viral genome encapsidation [225,278]; Viral genome cleavage and packaging [279]	Unknown
UL94	Negative regulator of the innate immune response (via MITA) [280]; Facilitates secondary envelopment [281]; Tegument protein, nucleocytoplasmic shuttling protein [282,283]; pp28 binding partner [284]; Interacts with UL99, protein localization, virus replication [285]	Immune evasion [280]
UL95	Late viral gene expression, infectious virus production [230]	Unknown
UL96	Nucleocapsid stability, maturation of the capsid dense bodies [286]	Unknown
UL97	Viral serine-threonine protein kinase, mimic cdc2/CDK1, DNA packaging [38,287]; Coregulates nuclear export of IFI16 [257,288,289]; Regulates IE gene expression by disrupting HDAC1 binding to the MIEP [290]; The cyclin-dependent kinase ortholog interacts with Cyclin T1 [291]; Involved in DNA replication, DNA encapsidation and/or nuclear egress [292,293,294,295]; Involved in secondary envelopment [296]; Subcellular distribution of cytoplasmic assembly sites [297]	Unknown
UL98	Alkaline nuclease [16,298,299,300,301]	Unknown
UL99 (PP28)	Myristylated phosphoprotein, secondary envelopment [3,281,302]; Interacts with UL94, protein localization, virus replication [284,285]; Intracellular trafficking, viral assembly [303,304]	Unknown
UL100	Virion glycoprotein (gM) [305]; gM/gN protein complex [306]; Virus assembly and replication [307]; Interacts with FIP4, recruits Rab11 [308]	Unknown
UL102	Component of DNA helicase-primase [38,309,310]	Unknown
UL103	Contributes to vAC biogenesis, secondary envelopment [160,311]; Antiviral responses, nuclear activities, biogenesis and transport of cytoplasmic vesicles [311]; Regulates virion and dense body egress [312]	Immune evasion [311];
UL104	DNA encapsidation, essential for DNA insertion into capsid [313,314,315,316]; Self-assemble portal complexes [317]; DNA packaging [318]	Unknown
UL105	Component of DNA helicase-primase [38,319]; Essential for oriLyt-dependent DNA replication [320]	Unknown
RNA5.0	Unknown	Unknown
UL108	Unknown	Unknown
UL109	Unknown	Unknown
UL110	Unknown	Unknown
UL111A	Homolog to hIL-10 [321,322]; Inhibits DC maturation and function [323]; Inhibits the recognition of latently infected cells by CD4^+^ T cells [324]; Immunomodulatory cytokine [325]; Correlates with the number of infiltrating T cells [326]; Enhances the CXCL12/CXCR4 signaling axis [327]; Upregulates hIL-10 expression, amplify its immunosuppressive impact [328]	Immune evasion [323,324,325,326,327,328]
UL112- UL113	Transcriptional activator [329]; Formation of replication compartment, efficient viral replication [320,330,331,332,333,334,335,336]	Unknown
UL114	Uracil DNA glycosylase (UNG), increases DNA synthesis efficiency [337,338,339]; Temporal regulation of DNA replication [340,341]; Interacts with UL54, participates in base excision repair [342]	Unknown
UL115	Virion glycoprotein L (gL), gH/gL/gO complex, cell fusion and entry [214,218,343]	Cell fusion [214]
UL116	Envelope glycoprotein, gH/UL116 complex, chaperone for gH [344,345]; Contributes to viral infectivity [346]; Interacts with UL148, promotes gH/gL complexes into virions [347]	Unknown
UL117	Promotes nuclear replication chamber development to facilitate viral growth [348]; Inhibits MCM accumulation, suppresses host DNA synthesis [349]	Unknown
UL119-118	gp68, Fcγ receptor, antagonizes host FcγR activation [350,351]; Carries IgGs and antibody MSL-109, interferes with IgG-mediated immunity [46,352,353]	Immune evasion [46,351,352,353]
UL120	Unknown	Unknown
UL121	Unknown	Unknown
UL122 (IE2)	Immediate early transactivator (IE2); interacts with transcriptional machinery; repression via specific DNA-binding activity [38]; Induces cells into S phase, alters cell cycle, induces cellular proliferation [34,190,354]; Induces TGF-beta expression [190,355]; Negative regulator MIEP [356]; Critical for replication [357]; Downregulates p53 activation [358]; Promiscuous transcriptional activator [359,360]; Regulates macrophage-mediated immune escape [361]	Cell proliferation [34] Immune evasion [361]
UL123 (IE1)	Immediate early transactivator (IE1); enhances activation by IE2; indirect effect on transcription machinery; disrupts ND10 [38]; Dysregulation of cyclin E expression; activation of telomerase; induction of IL-1; inhibition of apoptosis; induction of chromosomal aberrations [190]; Induces cells into S phase, alters cell cycle, conducive to proliferation [34,190]; Suppresses p53 and Rb activity, PI3K/AKT activation, induces cellular proliferation [33,190]; Limits nucleosome load, facilitates nucleosome reorganization, targets chromosomes [362,363]; Antagonize histone acetylation, facilitates viral replication [364]; Inhibits IFN-dependent STAT signaling [365,366,367]; Disruption of the dot-like structure of PML-NBs [368]	Cell cycle stimulation, cell proliferation [34,190] Immune evasion [365]
UL124	Unknown	Unknown
UL125	Unknown	Unknown
UL126A	Unknown	Unknown
UL127	No transcription [369,370,371,372]	Unknown
UL128	Chemokine analogue [373]; Induces cell PBMC proliferation and inflammation [32,374,375]; Viral entry and assembly, regulation of actin cytoskeleton [376]; Triggers monocyte paralysis, monocyte infection, blocks migration [377]; gH/gL/UL128-131 complex, promotes entry into cells, broadens virus tropism [214,378,379]	Cell proliferation, Induces inflammation [374] Cell migration [377] Cell tropism [380]
UL129	Unknown	Unknown
UL130	Viral entry and assembly [376]; gH/gL/UL128-131 complex, promotes entry into cells, broadens virus tropism [214,378,379]; pUL130 and Snapin interact to modulate DNA synthesis [381]; Cell tropism, promotes EC infection [382,383]	Cell tropism [382,383]
UL131A	Viral entry and assembly [376]; gH/gL/UL128-131 complex, promotes entry into cells, broadens virus tropism [214,378,379]; Important for endothelial cells tropism [384]; Virus entry and virus exit [385]	Cell tropism [384]
UL132	Regulates infectious virus production [386]; Viral glycoprotein, important for viral replication [387]	Unknown
UL148	Endoplasmic reticulum (ER)-resident glycoprotein, interacts with SEL1L [388,389]; Activates unfolded protein response [389]; ER reorganization, downmodulation of CD58, inhibits NK and T cell function [376,390,391,392]; Influences cell tropism by regulating gH/gL complex composition [393]	Cell tropism [392,393] Immune evasion [390,391]
UL147A	Downregulates MICA*008 to evade NK cell-mediated killing [394]	Immune evasion [394]
UL147	Ablate activity [376]; Viral CXC chemokine-2 (vCXCL2) [395,396,397]	Unknown
UL146	Ablate activity [376]; Viral CXC chemokine-1 (vCXCL1), induces calcium mobilization, chemotaxis, and degranulation of neutrophils [395,398]; Attracts neutrophils, influences viral dissemination [397,399]	Unknown
UL145	Constitutes vDCAF, impedes antiviral immunity [400]	Immune evasion [400]
UL144	Tumor necrosis factor-alpha (TNF-α) receptor [179,401]; Binds BTLA, inhibits T cell proliferation [37]; A potent NF-κB activator, evades immune surveillance [402,403]; HVEM orthologue, binds to B and T cell lymphocyte attenuator [404]	Anti-cell proliferation [37] Immune evasion [402,403]
UL142	Suppresses NK cell activation, inhibits NK cell killing [136,376,405]; Interferes with surface expression of full-length MICA alleles [406,407]; Interacts with Snapin [408]; Downregulates ULBP3, protects cells from NK cytotoxicity [409]	Immune evasion [406,407,409];
UL141	Regulates NK cell function (via TRAIL/CD155/CD112/ADCC) [47,136,376,410,411,412,413,414]; Interacts with CELF5, affects viral DNA replication [415]	Immune evasion [47,136,376,410,411,412,413,414]
UL140	Unknown	Unknown
UL139	Homologous to CD24, potential immunomodulatory role [416,417]	Unknown
UL138	Enhances modulation of TNF signaling [418,419]; Silences IE1 transcription, promotes viral latency [420,421,422,423]; Induces GC cells apoptosis by binding to HSP70 [424]; Modulation of EGFR signaling feeds back, represses virus replication [425,426,427]; Inhibits STING Pathway and reduces IFN-beta mRNA accumulation [428]; Interacts with USP1 activates STAT1 [429]	Cell apoptosis [424] Immune evasion [428]
UL136	Balances virus replication and latency [427,430,431]; Interacts with ATP1B1 [432]; Secondary envelopment and egress [433]; Golgi localization [434]; Postentry tropism in Endothelial Cells [435]; Regulates IL6/STAT3 pathway [436]	Cell tropism [435]
UL135	Postentry tropism in endothelial cells [435]; Suppresses formation of immunological synapse [65,437]; Promotes viral gene expression [438]; Regulates EGFR and reactivation [427,439,440]	Immune evasion [65,437];
UL133	Establishes latency, suppresses viral replication during latency [427,441,442]	Unknown
UL148A	Downregulates MICA to avoid NK cell attack [65,443]	Immune evasion [65,443]
UL148B	Unknown	Unknown
UL148C	Unknown	Unknown
UL148D	Unknown	Unknown
UL150	Unknown	Unknown
UL150A	Unknown	Unknown
IRS1	IE transcriptional activator [38,444]; Blocks shut down of translation [38,445]; Antagonizes PKR, facilitates virus replication [446,447]; Competitively associates with UL44 [448]; Causes AKT to remain active during infection [449]	Immune evasion [445]
US2	Prevents recognition by CD4 T cells [450]; Degradation of MHC-I, escapes recognition by T lymphocytes [451,452,453,454]; Interacts with hCD1d and downregulates iNKT cell activity [455]; Evades MHC- II antigen presentation [456]	Immune evasion [451,452,453,454]
US3	Degradation of MHC-I, evades MHC-II antigen presentation [456,457,458,459,460,461,462]	Immune evasion [456,457,458,459,460,461,462]
US6	Inhibits peptide translocation by TAP, degradation of MHC-I [461,463,464,465]	Immune evasion [461,463,464,465]
US7	US7 is modulated by miRNA [466]; Antagonize innate immunity by targeting TLR signaling [467]	Immune evasion [467]
US8	Antagonize innate immunity by targeting TLR signaling [467]; Binds MHC-I heavy chains [468]	Unknown
US9	Promotes cell-to-cell transmission [469]; Targets MICA*008 to escape NKG2D-mediated attack by NK cells [467,470]; Targets MAVS and STING signaling to evade innate antiviral response [471]	Immune evasion [467,470,471]
US10	NK cell activation [467]; Binds to MHC class I HC, delays MHC class I trafficking [472]; Degrades HLA-G to interfere with NK cell inhibition [473]	Unknown
US11	Suppresses MHC I-restricted recognition [451]; Degrades MHC-I, induce class I heavy chain destruction [474,475,476,477,478]; Escapes CD8+ T-cell immunity by degrading HLA-A and manipulating the HLA-B [479]	Immune evasion [451,474,475,476,477,478,479]
US12	NK cell evasion [480]; Induces autophagy via upregulating ULK1 phosphorylation and LC3-II conversion [481]	Immune evasion [480] Induces autophagy [481]
US13	Unknown	Unknown
US14	NK cell evasion function [480]	Immune evasion [480]
US15	Unknown	Unknown
US16	Virus tropism factor, regulates replication cycle [482,483,484]	Cell tropism [482,483,484]
US17	Control of virion composition to elicit a balanced host immune response [485]	Immune response [485]
US18	Putative transmembrane protein [486]; Downregulates B7-H6 surface expression to escape NK cell attack [480,487]; Promotes MICA degradation by lysosomal degradation [488]	Immune evasion [480,487,488]
US19	Putative transmembrane protein [486]	Unknown
US20	Putative transmembrane protein [486]; Downregulates B7-H6 surface expression to escape NK cell attack [480,487]; Promotes MICA degradation by lysosomal degradation [488]; Endotheliotropism, staged sustain replication cycle [489]	Immune evasion [480,487,488] Cell tropism [489]
US21	TMBIM-derived viroporin, modulates calcium homeostasis, protects cells against apoptosis [490]	Anti-apoptosis [490]
US22	Unknown	Unknown
US23	Unknown	Unknown
US24	Important for IE gene expression in replication cycle [491]	Unknown
US25	Unknown	Unknown
US26	Unknown	Unknown
US27	Chemokine receptor-like protein [103,492]; GPCR homologues, efficient spread by extracellular route [493]; Enhances the CXCL12/CXCR4 signaling axis [327,494,495,496,497,498]; Enhances cell proliferation [498,499]	Chemokine receptor [103] Immune evasion [494,496] Cell proliferation [498,499]
US28	Induces ADCC, immune evasion [47]; Chemokine receptor-like protein [103]; Mediated activation of NFκB and MIEP [228,500]; Promotes angiogenesis and tumor formation, oncomodulatory properties [501,502,503,504,505]; Promotes cell migration, fusion and viral dissemination, maintains latency [506,507,508,509]; Interacts with chemokines: CX3C and IL-8 [508,510]	Immune evasion [47] Chemokine receptor [103]
US29	Unknown	Unknown
US30	Unknown	Unknown
US31	Induces NF-κB-mediated mono-macrophage inflammation [511]; Activates immune response and regulates tumor immune microenvironment [512]	Induces inflammation [511,512]
US32	Unknown	Unknown
US33	Unknown	Unknown
US33A	Unknown	Unknown
US34	Unknown	Unknown
US34A	Unknown	Unknown
TRS1	Blocks shut down of translation [38,445]; Competitively binds UL44 [448]; Inhibit PKR activity, stimulates translation and replication [28,446,447,513,514]; Produces DNA-filled C-capsids, nuclear reorganization [515]; Inhibition autophagy [516]	Immune evasion [445] Inhibition autophagy [516]

**Table 2:** The information of HCMV lncRNAs during lytic infection.

LncRNA	Start	End	Length (bp)	Importance
RNA2.7	2218	4695	2477	Essential
RNA1.2	6368	7393	1025	Essential
RNA4.9	93,570	98,453	4883	Essential
RNA5.0	155,268	155,580	312	Essential
RNA5.0	160,112	160,944	832	Dispensable

Reference genome: KU926314.1.

**Table 3:** The information of conserved HCMV circRNAs during lytic infection.

HAN (KJ426589.1)	TB40E (KF297339.1)	Identity %	Alignment Length	Expectation Value
109,182|109,698	109,233|109,749	95	60	3.19 × 10^−23^
120,844|121,567	120,875|121,598	96.667	60	6.86 × 10^−25^
163,444|163,728	163,469|163,753	100	60	3.17 × 10^−28^
188,995|189,549	188,940|189,494	95	60	3.19 × 10^−23^
45,501|228,554	45,541|230,304	96.667	60	6.86 × 10^−25^
76,846|194,612	76,885|194,534	95	60	3.19 × 10^−23^
76,846|228,554	76,885|230,304	100	60	3.17 × 10^−28^
96,219|96,761	96,246|96,785	98.333	60	1.47 × 10^−26^
135,586|139,474	135,607|139,496	98.276	58	1.91 × 10^−25^
135,586|142,000	135,607|142,028	96.552	58	8.87 × 10^−24^
111,439|113,455	111,490|111,700	92.683	41	4.19 × 10^−12^
55,623|56,174	55,397|56,212	92.683	41	4.19 × 10^−12^

About 60 nt sequences around the back-splice junction points of the HAN and TB40/E strain circRNAs were compared using blastn (BLAST) [6].

## Data Availability

Not applicable.
